# Misleading Markers: Troponin-Positive Spontaneous Pneumomediastinum Masquerading as Acute Pericarditis in a Young Cannabis User

**DOI:** 10.7759/cureus.96425

**Published:** 2025-11-09

**Authors:** Aung Bhone Paing, Taha Elsahy, Abdullah Shaik, Dhruv Kundu, Maheen Iqbal

**Affiliations:** 1 Acute Medicine, Peterborough City Hospital, Peterborough, GBR; 2 General Medicine, Peterborough City Hospital, Peterborough, GBR

**Keywords:** cannabis, chest pain, pericarditis, pneumomediastinum, smoking, troponin

## Abstract

Spontaneous pneumomediastinum (SPM) is defined as the presence of free air within the mediastinum without an apparent precipitating cause such as trauma, invasive procedures, or oesophageal rupture. SPM is typically benign and self-limiting but may mimic life-threatening conditions such as myocardial infarction, pulmonary embolism, or aortic dissection. The pathophysiology is most explained by the Macklin effect, where increased intra-alveolar pressure leads to alveolar rupture and dissection of air along bronchovascular sheaths into the mediastinum. Recognized risk factors include intense physical exertion, vomiting, Valsalva manoeuvres, and recreational drug use such as cannabis and cocaine inhalation. Although SPM usually follows a benign course, prompt diagnosis is important to exclude secondary causes such as Boerhaave syndrome or tracheobronchial injury, which carry significant morbidity and mortality if missed. We report the case of a healthy 18-year-old male who developed extensive spontaneous pneumomediastinum shortly after smoking, with a background of prior cocaine use. This case highlights the importance of recognizing SPM in the differential diagnosis of acute chest pain in young adults and reinforces the value of conservative management following thorough exclusion of secondary causes.

## Introduction

Pneumomediastinum, also known as mediastinal emphysema, is an unusual clinical presentation characterized by the presence of free air within the mediastinal cavity. It typically results from alveolar rupture secondary to a sudden increase in intra-alveolar pressure, allowing air to dissect along the bronchoalveolar sheath into the mediastinum [1]. 

Spontaneous pneumomediastinum (SPM) most often affects young males and commonly presents with chest pain, neck pain, and dyspnea. It is frequently associated with activities that cause abrupt rises in intrathoracic pressure, such as forceful coughing, vomiting, Valsalva maneuvers, intense physical exertion, or, in rarer cases, cannabis smoking [1,2]. The association between marijuana use and SPM is thought to stem from the characteristic inhalation techniques adopted by users, prolonged breath-holding, deep inhalation, and repetitive Valsalva-like manoeuvres. It can lead to marked fluctuations in intrathoracic pressure and subsequent alveolar rupture [2,3]. 

On physical examination, patients may exhibit tachycardia, tachypnea, and hoarseness of voice. Subcutaneous emphysema is observed in approximately 35-58% of cases, while the more specific Hamman’s sign, a characteristic crunching sound synchronous with the heartbeat, is present in about 6-18% [1]. Diagnostic evaluation typically involves chest radiography and computed tomography (CT) to confirm mediastinal air and exclude other life-threatening differentials such as esophageal perforation or pneumothorax [1,4]. Although SPM generally follows a benign and self-limiting course, potential complications can be severe; hence, comprehensive initial assessment and appropriate monitoring are essential to ensure favorable outcomes [1,5] 

## Case presentation

An 18-year-old male presented with a history of sudden-onset chest pain that developed while he was at work. At the time of onset, he was smoking a cigarette. The chest pain gradually worsened over the following two hours and was associated with a severe tightening sensation on the right side of the neck and throat, shortness of breath, and pain exacerbated by deep inspiration. He denied fever, upper respiratory tract symptoms, rash, gastroesophageal reflux, abdominal pain, trauma, or recent illness. 

The patient reported smoking five to six cigarettes daily, occasional cannabis use, and a single episode of cocaine use three months prior. He had no significant past medical history and was not taking any regular medications. On examination, the patient was alert, comfortable, and seated upright. There was no lymphadenopathy, and the trachea was central. Lung sounds were clear bilaterally. A crunching sound synchronous with the heartbeat (Hamman’s sign) was noted on auscultation. Cardiovascular examination revealed no murmurs, no raised jugular venous pressure, and no signs of trauma. There was no palpable subcutaneous crepitus. 

Vital signs were stable: respiratory rate 20 breaths/min, oxygen saturation 98% on room air, blood pressure 134/86 mmHg, heart rate 80 beats/min, and temperature 36.1 °C. Investigations showed a normal 12-lead ECG except for T-wave flattening in lead aVL (serial ECGs were also done to ensure there were no dynamic changes). Laboratory test results are presented in Table 1. Serial troponins (four hours apart each) were 37, 30, and 15 ng/L. Laboratory results revealed a D-dimer of 118 ng/mL, white cell count (WCC) 11.1 × 10⁹/L, C-reactive protein (CRP) 1 mg/L, and neutrophils 8.3 × 10⁹/L.

**Table 1:** Laboratory Findings Troponin: Cardiac biomarker indicating myocardial injury; D-dimer: Marker of fibrin degradation, used to assess for thromboembolism; White Cell Count (WCC): Total count of white blood cells, indicating immune response; C-Reactive Protein (CRP): Marker of inflammation; Neutrophils: Subtype of white blood cells, elevated in infection or inflammation.

Test	Patient Values	Units	Reference Ranges
Troponin (1st)	37	ng/l	<14 ng/l
Troponin (2nd)	30	ng/l	<14 ng/l
Troponin (3rd)	15	ng/l	<14 ng/l
D-Dimer	118	ng/ml	<500 ng/ml
White Cell Count	11.1	× 10⁹/l	4.0–11.0 × 10⁹/l
C-Reactive Protein	1	mg/l	<5 mg/l
Neutrophils	8.3	× 10⁹/l	2.0–7.5 × 10⁹/l

**Figure 1:**
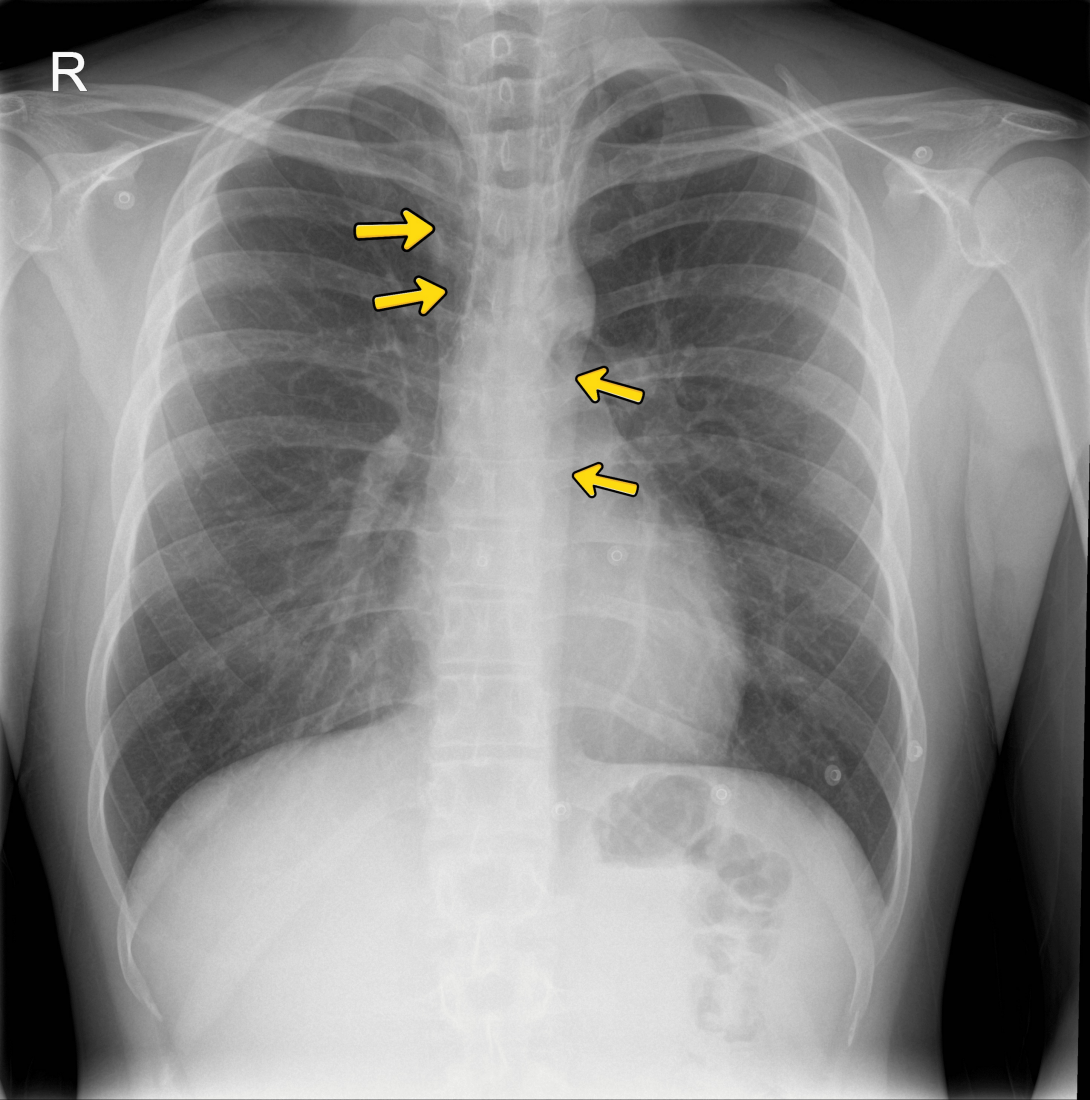
Chest radiograph showing pneumomediastinum

Chest radiography (Figure 1) demonstrated pneumomediastinum in the upper mediastinal region, with clear lung fields and no cardiomegaly. 

**Figure 2:**
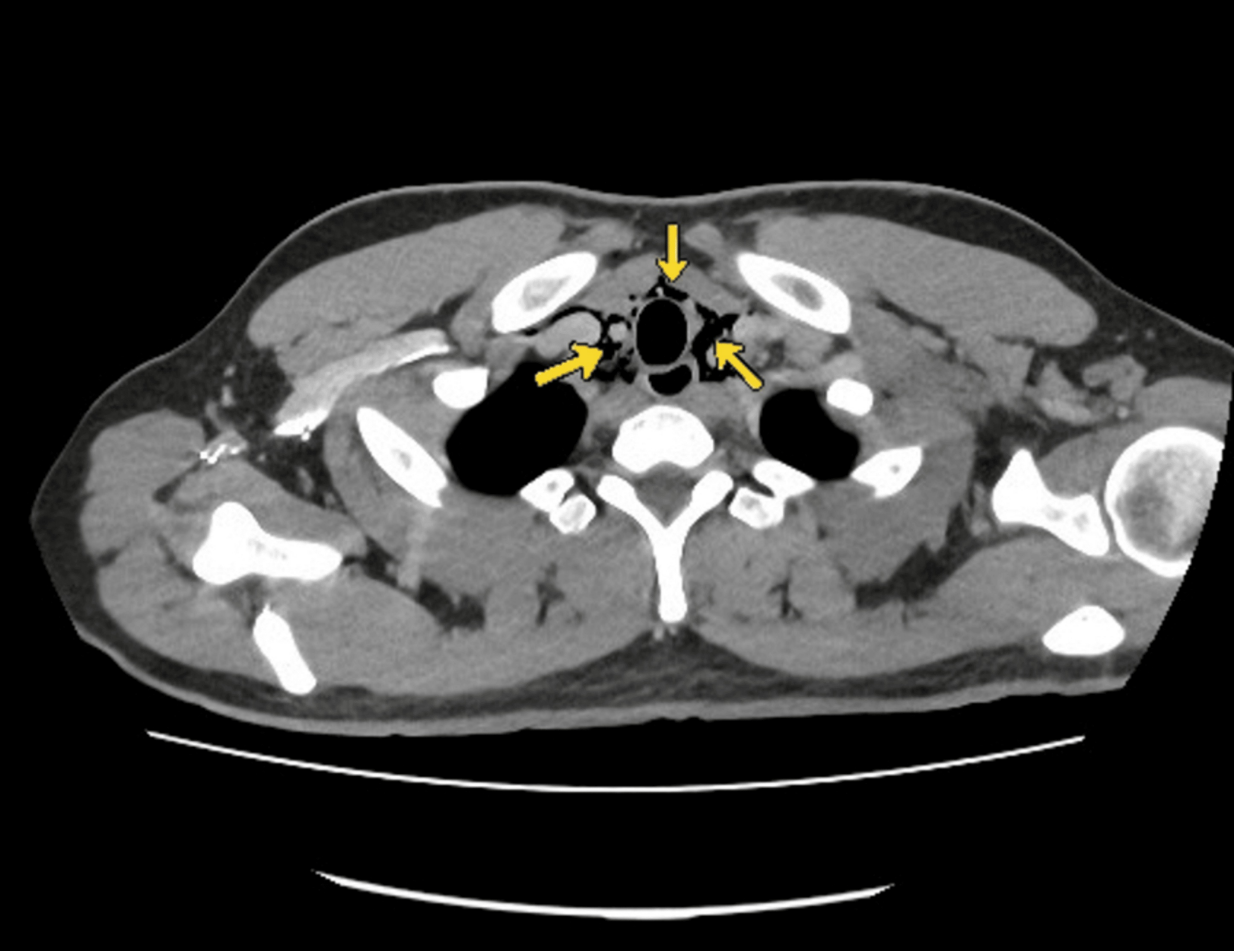
CT Chest with oral contrast showing pneumomediastinum

**Figure 3:**
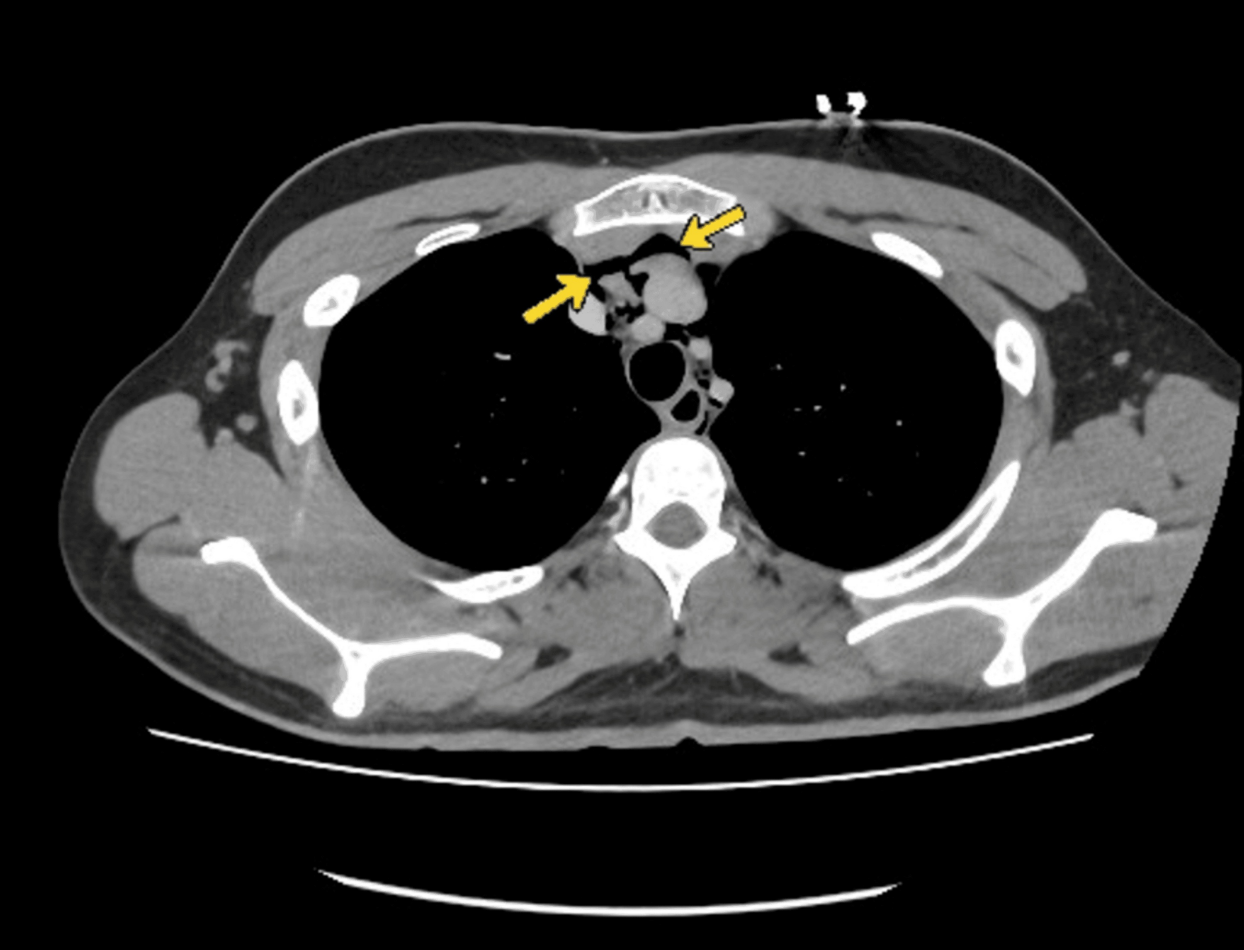
CT Chest with oral contrast showing pneumomediastinum

CT of the neck and thorax with contrast (Figures 2, 3) confirmed extensive spontaneous pneumomediastinum extending into the cervical soft tissues, with no identifiable underlying cause. 

**Figure 4:**
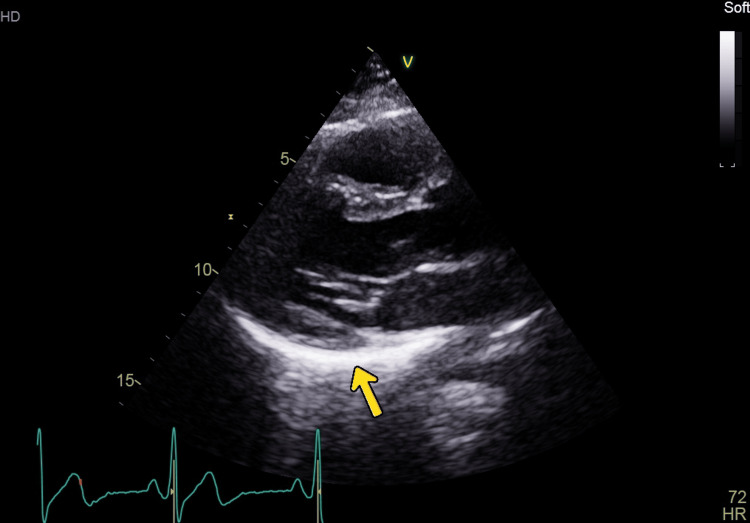
Echocardiogram suggestive of pericarditis

The patient was managed conservatively with analgesia. The cardiothoracic team advised observation for 24 hours, with no surgical intervention required. Echocardiogram (Figure 4) revealed a bright, thickened posterior pericardium consistent with possible acute pericarditis, but without pericardial effusion or functional compromise. Left ventricular systolic function was at the lower end of normal (EF 54%), with normal right ventricular and valvular findings.

The cardiology team considered the echocardiographic features likely related to surrounding air rather than true pericarditis, given the atypical clinical presentation, normal serial ECGs, and down-trending troponin. The patient remained clinically stable, was deemed medically optimized, and was discharged with analgesia and routine outpatient cardiology follow-up.

## Discussion

The cause of pneumomediastinum can be extra-thoracic (e.g., chest trauma) or intrathoracic (e.g., esophageal rupture) [6]. However, sometimes it can also occur spontaneously without any apparent cause (SPM). It is also known as Hamman’s syndrome, named after the American physician and diagnostician, Louis Virgil Hamman, who first published a case series on it in 1939 [7]. SPM is an uncommon cause of chest and neck pain that frequently gets over-investigated due to its ambiguity, often mimicking more sinister causes [8]. Our case report highlights its complexity, potential for misdiagnosis, and the possible link to cannabis use. 

One of the hypotheses of cannabis use being associated with SPM is the method of inhalation. Deep inhalation with prolonged breath holding (Valsalva manoeuvre) and repeated forced inhalation can increase intra-thoracic and intra-alveolar pressure, making the alveoli susceptible to rupture. Another possible cause is excessive vomiting caused by cannabis use, which can also induce the Macklin effect [2]. 

Although the link has not been confirmed, there have been reports of cases where pneumomediastinum causes elevation of troponin [9] and ST changes in ECG occasionally [10]. However, it is vital to exclude other causes of these changes, which may be coexisting with SPM, such as acute coronary syndrome or pulmonary embolism. The Hamman’s sign is pathognomonic but could also be mistaken for a pericardial rub due to the uncommon nature of SPM.

In terms of imaging, one could look for radiological signs like the ''Ring around the Artery'' sign and the ''Continuous Diaphragm sign'' [11]. Echocardiograph findings can also mimic an acute pericarditis, as seen in this case. 

Although SPM usually follows a benign course, it is crucial to rule out secondary causes of pneumomediastinum such as Boerhaave syndrome. The mainstay management is conservative, but it is important to observe for signs of respiratory distress, since approximately 10% of patients with SPM also have pneumothorax [12]. 

There is no exact recommended guideline on when to follow up, as SPM is self-limiting. The clinician would need to decide on a case-by-case basis, depending on the clinical condition.

## Conclusions

The clinician should consider pneumomediastinum when assessing a young patient with possible pericarditis. Elevation of troponin does not always point to an issue of cardiac origin. Clues such as presence of Hamman’s sign and history of cannabis use may shine a light on the correct diagnosis, although more research is needed to confirm the latter. 
